# Methods, applications, and computational challenges in bait capture enrichment

**DOI:** 10.1016/j.crmeth.2025.101174

**Published:** 2025-09-15

**Authors:** Jonathan E. Bravo, Kimberly J. Newsom, Noelle Noyes, Christina Boucher

**Affiliations:** 1 Department of Computer and Information Science and Engineering, Herbert Wertheim College of Engineering, University of Florida, Gainesville, FL, USA; 2 Department of Pathology, Immunology and Laboratory Medicine, University of Florida, Gainesville, FL, USA; 3 Department of Veterinary Population Medicine, University of Minnesota, St. Paul, MN, USA

**Keywords:** targeted sequencing, hybridization capture, antimicrobial resistance, viromics, pathogen detection

## Abstract

Bait capture enrichment techniques have revolutionized our understanding of complex biological systems by enabling the selective isolation of specific genomic regions for detailed study. This review offers a comprehensive examination of bait capture enrichment, evaluating its advantages, limitations, and applications. We explore the computational challenges inherent in bait capture enrichment, including bait design, deduplication, variant detection, and the modeling of off-target binding. Current solutions and open problems in these areas are discussed, highlighting potential future research directions. By addressing these challenges and improving bait capture methodologies, we can enhance the ability to investigate genomic regions of interest with greater precision and efficiency, ultimately advancing our understanding of fundamental genetic and biological processes.

## Teaser

Bravo et al. review bait capture enrichment methods for targeted genomic sequencing, systematically examining applications across bacterial genomics, viral surveillance, and antimicrobial resistance detection. They discuss computational challenges and potential solutions toward enhanced target recovery from complex samples.

## Introduction

The challenge of detecting and analyzing nucleic acid sequences from low-abundance organisms within complex and diverse samples has been a persistent obstacle in molecular biology. Early efforts to overcome this limitation can be seen in the development of Southern blotting in 1973^1^ and the invention of polymerase chain reaction (PCR) in 1985,^2^ both of which improved detection of specific low-abundance nucleic acid sequences in complex samples. Variations of these methods have been developed over time, including the use of multiplex PCR- and biotinylated probe-based assays, which can detect a large number of nucleic acid sequence targets in one workflow. Bait capture enrichment, also known as target or hybrid capture, is a well-established technique in molecular biology and genomics. This method employs baits that are short, single-stranded DNA or RNA oligonucleotides designed to hybridize with complementary target sequences. By using these baits in a complex sample, specific genomic regions can be selectively captured and then amplified, even when these targets are present at very low abundance or within a high background of non-target DNA.^3^^,^^4^ Baits typically range from 60 to 150 base pairs, and are longer than conventional PCR primers. Baits are generally more tolerant of genetic variation than PCR primers and thus can often capture highly variable targets, such as rapidly mutating viruses.

**Figure 1:**
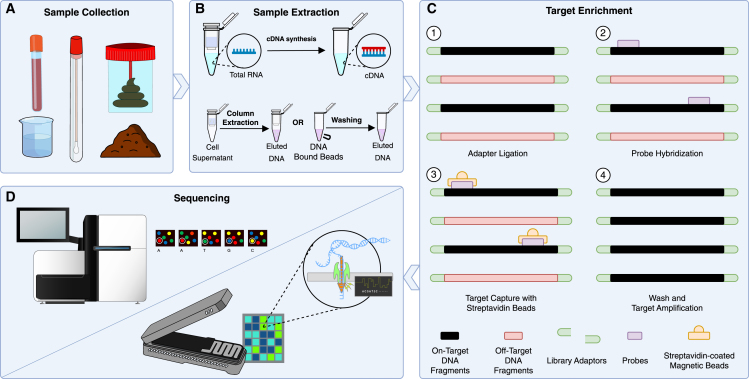
Illustration of the laboratory workflow for bait capture enrichment (A) This workflow outlines key steps in bait capture. Sample collection includes gathering fluids, tissues, or substrates. (B) DNA or RNA extraction follows, with RNA undergoing cDNA synthesis. DNA is then fragmented to appropriate sizes for capture. (C) After (1) adapter ligation and PCR amplification, (2) specific genomic regions are captured using probes designed to hybridize with target sequences. (3) The DNA is then enriched through a sequence of probe hybridization and capture on streptavidin-coated magnetic beads, which selectively bind to biotinylated probes attached to the on-target fragments, allowing for the (4) washing away of non-target fragments. (D) The enriched libraries are sequenced, facilitating detailed analysis of the targeted and potentially flanking regions.

Bait capture enrichment begins with bait design, a bioinformatics process where oligonucleotide sequences are computationally selected and optimized for target capture. Following bait design and manufacture, the experimental workflow (Figure 1) begins with sample collection (A) and DNA or RNA extraction (B). Preparation of a sequencing library from the resulting nucleic acid follows, beginning with adapter ligation (C1). Following library preparation, the enrichment process begins with denaturation of the library and hybridization with biotinylated probes, enabling capture of targeted genomic regions (C2). These bait-target complexes are then isolated using streptavidin-coated magnetic beads (C3), and a series of washes removes non-target fragments (C4). Finally, the enriched libraries are sequenced to generate the final data (D). We note that bait panels can be large, allowing for the capture and selective amplification of megabases of genomic sequence in a single experiment, including entire gene families or large chromosomal regions.^5^ Hence, bait capture enrichment offers a strategic alternative to both culture-dependent sequence workflows such as whole-genome sequencing (WGS) and to culture-independent sequence workflows such as metagenomics.^6^ By concentrating sequencing efforts on specific genomic loci within and/or across diverse genomes, bait capture enrichment enables greater sequencing depth and/or a larger number of samples to be multiplexed, thereby enhancing throughput.^7^^,^^8^ This enhanced throughput—or enrichment—is commonly measured as on-target percentage, representing the fraction of sequencing reads that successfully capture the intended genomic regions. This concentration of informative data is important for applications demanding high confidence, such as clinical-grade variant calling and haplotyping.^9^ Even when dealing with challenging samples like ancient DNA or complex metagenomes, bait capture can effectively isolate desired sequences. While the necessary trade-off is the exclusion of non-targeted genomic regions,^10^ bait capture enrichment assists in the interrogation of detailed genetic information and the elucidation of biological functions within specific, user-defined genomic contexts across a wide range of research fields.

Despite its advantages, bait capture enrichment involves inherent complexities and potential limitations such as increased workflow duration to allow for the selective binding of baits to targets and selection biases introduced during capture and amplification.^11^ A comprehensive overview of bait capture enrichment methods, an evaluation of the advantages and limitations, and an exploration of the specific applications in various fields are presented herein. We delve into the computational challenges associated with bait design, processing enriched sequencing data, and managing off-target sequences. By examining current solutions and potential future directions such as protocol optimizations, depletion strategies, and integration with novel technologies, we aim to provide a clear perspective on the current state and future potential of bait capture enrichment for advancing genomic research.

## Applications of bait capture enrichment

Bait capture enrichment has proved particularly valuable for studying rapidly evolving biological systems, where the method’s tolerance for genetic variation and capacity to capture targets at low abundance provide some advantages over conventional sequencing approaches. The technique’s effectiveness stems from how baits function at the molecular level: baits bind to their targets within a longer contiguous strand of nucleic acid, and they are able to capture both the targeted and adjacent regions, enabling the detection and characterization of flanking sequencing, including nearby variants and structural features.^12^ This capability is particularly useful in studying highly divergent or rapidly evolving loci, such as those associated with horizontal gene transfer,^13^ antimicrobial resistance (AMR),^14^ pathogenicity,^15^ and cancerous cell lines.^16^ In this section, we describe a number of these applications in more detail.

### Targeted bacterial enrichment

In microbial genomics, bait capture enrichment is increasingly utilized to recover bacterial pathogen genomes without the need for prior culture. Bait enrichment can increase detection sensitivity and enable in-depth genomic analysis from diverse sample types.^17^^,^^18^ By increasing the relative concentration of target bacterial DNA compared to background DNA, bait capture enrichment improves detection thresholds for downstream molecular assays, including both sequencing and qPCR-based methods, often enabling the recovery of more complete bacterial genome sequences.^17^^,^^18^ The selective isolation of bacterial DNA offers an alternative, when traditional culture-based methods fail to yield results, as can be the case for bacterial pathogens that are difficult to grow due to specific metabolic needs, those currently deemed unculturable, or those classified as hazardous.^17^^,^^19^ For bacterial research, bait capture enrichment enhances the efficiency of pathogen detection and enables more comprehensive genomic analyses.

The culture-independent nature of bait capture enrichment is particularly relevant for public health microbiology when investigating bacterial pathogens that are difficult or impossible to culture.^12^^,^^17^ For instance, surveillance and diagnosis of bacterial sexually transmitted infections (STIs) caused by organisms like *Neisseria gonorrhoeae*, *Treponema pallidum*, *Mycoplasma genitalium*, and *Chlamydia trachomatis* have historically been constrained by culture limitations.^19^ These difficulties are often resource dependent, leading to potential biases in surveillance data, which may underrepresent infections in remote or less-funded regions.^19^ Bait capture enrichment, coupled with sequencing, circumvents the need for culture, enabling comprehensive genomic analyses, including high-resolution strain typing and phylogenetic analysis, directly from patient specimens.^19^ Examples include the application of bait capture enrichment to sequence *T. pallidum* directly from clinical samples to track the emergence and spread of macrolide resistance across different lineages^20^ and its combination with Nanopore sequencing for culture-independent detection of *N. gonorrhoeae* and its AMR determinants directly from clinical samples.^21^ While challenges related to cost, workflow standardization, and bioinformatics persist, bait capture enrichment’s continued development and use are promising for addressing gaps in understanding transmission dynamics and molecular epidemiology of these pathogens.^19^

The practical application of bait capture enrichment in bacterial genomics was advanced by the development of specific protocols targeting key pathogens directly from complex sample matrices. For instance, Christiansen et al.^22^ first demonstrated the feasibility of using custom RNA baits for direct whole-genome enrichment (WGE) and sequencing of *C. trachomatis* from clinical samples, achieving a greater than 10-fold higher sensitivity than other methods. Subsequent work by Bowden et al.^23^ further refined this approach for *C. trachomatis* with a publicly accessible bait library, yielding an average 20% increase in classified reads. This strategy was also successfully applied to other pathogens; for example, Verma et al.^24^ employed RNA-bait hybrid capture to enrich and sequence *Mycobacterium tuberculosis* (Mtb) DNA directly from environmental samples, achieving a roughly 3× increase in median coverage, demonstrating the effectiveness of bait capture enrichment for environmental surveillance. Complementing this, Brown et al.^25^ enriched for Mtb WGS data with a mean coverage increase from 4.6× to 200×, directly from clinical sputum samples.

Building upon these established successes, bait capture enrichment has proved its versatility and effectiveness across an even broader range of challenging bacterial targets and diverse sample types. For example, Dennis et al.^17^ successfully used bait capture to obtain WGS data for the difficult-to-culture *Mycoplasma amphoriforme* and the hazardous *Bacillus anthracis* from clinical and environmental samples; Paskey et al.^18^ applied bait capture enrichment to enhance both qPCR and WGS detection of *rickettsial* pathogens (*Rickettsia prowazekii*, *Orientia tsutsugamushi*) from clinical specimens; Carpi et al.^26^ captured *Borrelia burgdorferi* genomes directly from tick samples; and Schuenemann et al.^27^ used DNA probe capture to reconstruct ancient *Mycobacterium leprae* genomes from highly degraded DNA from medieval human remains, offering insights into the historical evolution of leprosy. Table 1 summarizes these and other studies that use bait capture enrichment to recover bacterial genomes, showing key innovations and applications.

**Table 1:** Use of bait capture enrichment to recover bacterial genomes

Technology/approach	Year	Description
Ancient *M. leprae* WGE	2013	pioneering use of solution-based DNA probe capture to reconstruct ancient *M. leprae* genomes from highly degraded medieval skeletal remains, enabling paleomicrobiology investigations^27^
*C. trachomatis* WGE	2014	landmark application demonstrating feasibility of custom RNA baits for direct WGE and sequencing of *C. trachomatis* from clinical samples, significantly increasing sensitivity over prior methods^22^
*M. tuberculosis* environmental WGE	2022	novel application of RNA-bait hybrid capture for environmental surveillance, enriching and sequencing *M. tuberculosis* DNA directly from environmental surface swabs^24^
Rickettsial WGE and qPCR enhancement	2024	demonstrated that bait capture enrichment enhances sensitivity for both WGS- and qPCR-based detection of rickettsial pathogens (*R. prowazekii*, *O. tsutsugamushi*) directly from clinical specimens^18^
*N. gonorrhoeae* WGE + Nanopore	2024	integration of bait capture enrichment with long-read Nanopore sequencing for culture-independent WGS and AMR determinant detection of *N. gonorrhoeae* directly from clinical samples^21^

This table highlights landmark studies and approaches demonstrating the application of bait capture enrichment for obtaining whole or near-whole bacterial genomes directly from challenging samples. Examples include methods developed for reconstructing ancient pathogen genomes (e.g., *M. leprae*), sequencing fastidious bacteria directly from clinical specimens (e.g., *C. trachomatis*, *N. gonorrhoeae*), environmental surveillance (e.g., *M. tuberculosis*), and enhancing detection of vector-borne pathogens (e.g., rickettsiae). These techniques typically utilize hybridization capture with custom-designed baits based on reference genomes to overcome issues like low pathogen abundance or DNA degradation, significantly enhancing sensitivity for sequencing and diagnostics, sometimes in combination with qPCR or long-read sequencing.

### Targeted viral enrichment

Sequencing only viral genomes, especially multiple distinct viruses co-existing within biological samples, presents challenges for viral surveillance and discovery,^28^^,^^29^ and, despite considerable advancements in molecular techniques, limitations persist.^30^ Traditional PCR-based diagnostics, though sensitive and specific for known targets, depend on precise sequence information for primer design and binding. This requirement for exact complementary matches restricts PCR’s utility in discovering novel viruses or sequencing variants with high mutation rates.^28^^,^^31^^,^^32^ Metagenomic sequencing offers a broader, culture-independent alternative, theoretically enabling the detection of all genetic material within a sample.^33^^,^^34^ However, its practical application for viromics is often hampered by low sensitivity, particularly when the sheer volume of host or background microbial DNA obscures low-abundance viral DNA, making viral detection and genome assembly difficult, as is common in host-dominated clinical or environmental samples.^35^^,^^36^

**Figure 2:**
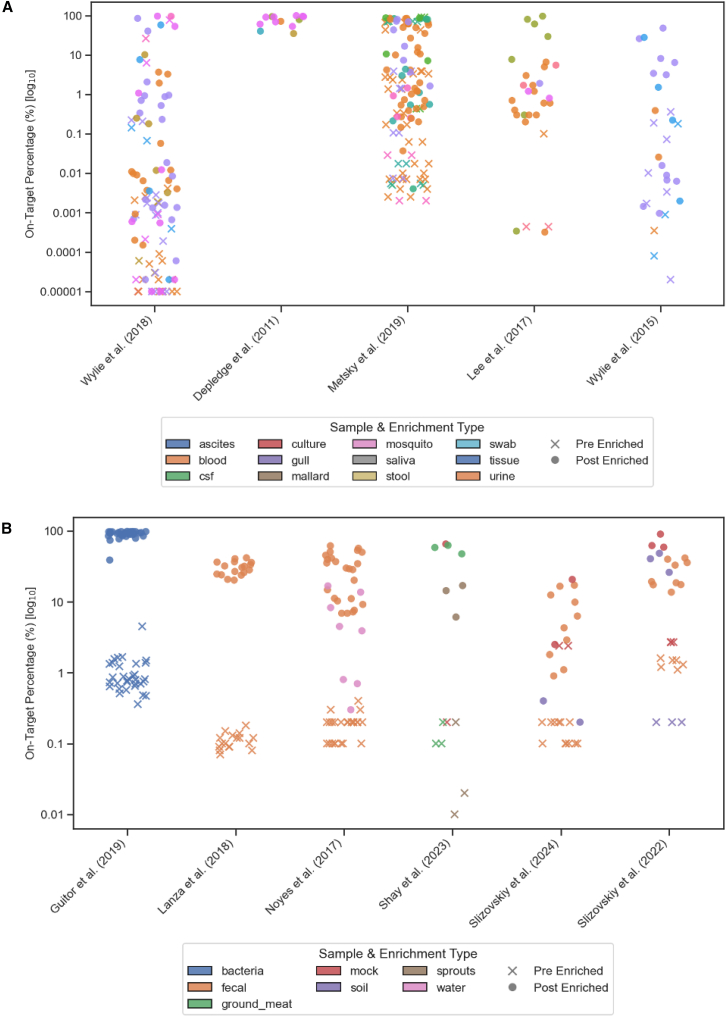
On-target percentage before and after enrichment by source and sample type (A) Scatterplots illustrate on-target percentages, in log_10_ scale, for whole viral capture, from previously published studies^33^^,^^34^^,^^35^^,^^39^^,^^40^ for multiple samples before (cross markers) and after (dot markers) enrichment, categorized by sample type. These plots emphasize the impact of enrichment techniques in improving on-target percentages, thus enhancing data quality, particularly in samples with low-abundance targets. (B) Scatterplots illustrate on-target percentages, in log_10_ scale, for gene capture, from previously published studies^3^^,^^4^^,^^41^^,^^42^^,^^43^^,^^44^ for multiple samples before (cross markers) and after (dot markers) enrichment, categorized by sample type. These plots emphasize the impact of enrichment techniques in improving on-target percentages, thus enhancing data quality, particularly in samples with low-abundance targets.

Bait capture enrichment directly addresses these limitations by selectively capturing viral DNA prior to sequencing through hybridization,^12^ to significantly increase the proportion of viral reads in the final sequencing data (Figure 2A). Biotinylated baits are designed to hybridize to known or conserved regions of target viral genomes. These captured viral fragments are then isolated, often using streptavidin beads, and amplified, effectively enriching the viral signal while reducing the background of host or other non-target sequences. A key advantage of this approach, particularly relevant for diverse RNA viruses, is its tolerance for sequence mismatches between the bait and the target sequence.^32^^,^^37^ This allows for the capture not only of known viruses but also of related, divergent strains or even novel viruses within targeted families,^37^ as seen with the discovery and characterization of bat-borne coronavirus, identified using custom baits.^38^

The practical utility of bait capture enrichment in virology is underscored by numerous applications that effectively enhance viral detection, genomic characterization, and discovery in complex samples. For instance, broad-spectrum approaches like the ViroCap method improve virome sequencing sensitivity against standard metagenomic approaches by successfully identifying low-abundance viruses and capturing sequences with up to 58% divergence from bait references.^39^ Similarly, the compact aggregation of targets for comprehensive hybridization (CATCH) method employed comprehensive probe sets for maximal diversity targeting 356 human-infecting viral species, enabling the assembly of previously undetectable genomes, and has proved effective in challenging scenarios, such as Lassa virus recovery during an outbreak.^40^ The importance of rigorous clinical validation for such broad panels is highlighted by systems like VirCapSeq-VERT, which demonstrate diagnostic accuracy comparable to qPCR for many viral targets.^45^

Beyond broad surveillance, bait capture enrichment is readily adapted with custom panels for specific pathogens, viral families, or research questions, often yielding quantitative gains. Lee et al.,^35^ applied targeted genome capture (TGC) for feline pathogens, achieving enrichment factors from 58-fold up to 56 million-fold relative to host DNA, facilitating robust taxonomic classification, though performance notably varied with sample quality. Custom panels have also been used for targeted outbreak surveillance, such as the filovirus panel developed by Koehler et al.,^46^ and for investigating specific viral reservoirs. Illustrating the latter, Li et al.^47^ used custom baits to identify diverse bat coronaviruses often missed by traditional methods due to high background signal, demonstrating more efficient use of sequencing resources and reduced data analysis burdens. These and other targeted viral enrichment technologies are summarized in Table 2.

**Table 2:** Overview of selected bait capture enrichment technologies and protocols for the study of viruses

Technology	Year	Description
Solution hybrid selection	2009	foundational technique using ultra-long biotinylated RNA baits for massively parallel targeted sequencing of genomic regions (adapted later for viruses)^12^
Filovirus capture panel	2014	panel of sequence capture probes designed to detect multiple filoviruses (Ebola, Sudan, Reston, Taï Forest, Bundibugyo, Marburg variants) using NGS^46^
ViroCap	2015	bait capture enrichment designed to enrich nucleic acid from DNA and RNA viruses from 34 families known to infect vertebrates, enhancing sensitivity and detection of divergent strains^39^
TGC	2017	application of bait capture enrichment demonstrating high fold enrichment (up to 56 million-fold) for specific feline pathogens relative to host nucleic acids^35^
CATCH	2019	CATCH: a computational method for designing optimal, comprehensive, and scalable probe sets, validated with a panel for 356 human viral species^40^
Bat CoV capture	2020	capture-based NGS using custom-designed baits targeting a broad range of coronaviruses, enabling discovery and characterization from bat samples with high background noise^47^
TELSVirus	2023	integration of bait capture enrichment with long-read sequencing for enhanced detection and full viral genome reconstruction^48^

This table summarizes key technologies and panels developed for enriching viral nucleic acids from complex biological samples prior to sequencing. Featured approaches range from foundational methods like solution hybrid selection to broad-spectrum panels designed for comprehensive vertebrate virus detection (e.g., ViroCap), specific viral groups (e.g., Filovirus panel), computationally optimized probe design methods (e.g., CATCH), and techniques integrated with long-read sequencing (e.g., TELSVirus). These methods generally employ hybridization capture, using baits designed against known or conserved viral sequences, to significantly improve detection sensitivity, facilitate more complete genome assembly, and enable discovery or surveillance of known, divergent, or even novel viruses within complex backgrounds.

Despite these successes, early applications often relied on short-read sequencing, which can complicate full-length genome reconstruction and phased variant analysis, limiting insights into viral diversity and co-infection dynamics.^33^^,^^34^^,^^40^^,^^49^ While a need remains for more standardized performance metrics and direct comparisons across diverse panels and sample types,^50^ recent innovations are addressing these limitations. The combination of enrichment with long-read sequencing technologies, supported by early feasibility studies^10^ and now implemented in dedicated workflows like TELSVirus,^48^ which enhances the ability to reconstruct complete viral genomes from complex samples and is useful for studying co-circulating viruses, tracking evolution, understanding transmission, and performing phased variant analysis, represents an advancement for comprehensive viral surveillance and characterization.

### Targeted gene enrichment

Beyond the genomic analysis of whole organisms, sequencing efforts frequently target specific genes or gene families associated with important phenotypes, such as AMR, virulence, or desirable agricultural traits like pathogen resistance or crop quality. Studying these genes directly within complex samples is often challenging.^51^ Traditional PCR-based methods offer sensitivity for previously defined targets; however, they often prove less effective when applied to highly diverse gene families or for the detection of novel variants.^52^ Conversely, shotgun metagenomic sequencing provides an unbiased view but often lacks the sensitivity to detect low-abundance genes or provide sufficient coverage for detailed analysis, as target genes might constitute less than 1% of the total sequence data.^51^ This limitation is particularly problematic for targets like antibiotic resistance genes (ARGs), where even rare variants or those present in minor microbial populations can be clinically significant but are easily overlooked by standard metagenomic approaches.^3^^,^^4^

Bait capture enrichment offers a solution—to enrich specific genes or gene families directly from complex DNA pools, increasing the sensitivity of sequencing for targeted gene studies.^41^^,^^42^ When incubated with a fragmented DNA library, baits will selectively hybridize to the targeted gene fragments. These captured fragments are then isolated and sequenced, resulting in a dataset highly enriched for the genes of interest^42^^,^^53^ (Figure 2B). Tolerance for mismatches during hybridization allows baits to capture related but divergent alleles or members of large gene families.^41^^,^^54^

Enrichment enhances the detection of ARGs, which often constitute a small fraction of metagenomic data. Early work by Lanza et al.^4^ (ResCap platform) and Noyes et al.^3^ (MEGaRICH platform) demonstrated that bait capture could identify hundreds of additional ARGs, including low-abundance but clinically important genes, like extended-spectrum beta-lactamases (ESBLs) and carbapenemases, which were missed by conventional shotgun sequencing. These studies highlighted that the low-abundance resistome differs compositionally from the high-abundance portion of the resistome and contains significant genetic diversity (e.g., SNPs) that can be used to discriminate between sample types. Subsequent studies utilized baits designed from comprehensive databases like the Comprehensive Antibiotic Resistance Database (CARD)^55^ to explore ARG acquisition in specific contexts, such as *Salmonella* in the chicken gut^56^ or across diverse bacterial types and human stool samples. Guitor et al.^41^ demonstrated the robustness of the approach, achieving high coverage of targeted ARGs even for short gene fragments across different library preparation methods. Enrichment has also proved valuable in food safety surveillance, significantly increasing ARG detection rates (e.g., >300-fold^42^ or 40-fold^53^) in retail food samples compared to non-enriched data, helping to identify potential ARG reservoirs.

While identifying ARG reservoirs is a key outcome of enhanced detection, the public health risk posed is largely determined by the mobility potential of the ARGs they contain. Resistance can spread rapidly via mobile genetic elements (MGEs) like plasmids and transposons, making it crucial to understand these ARG-MGE associations. Although short-read sequencing, following enrichment, boosts ARG detection, it struggles to link ARGs to the surrounding genomic context, including MGEs, due to assembly fragmentation issues, particularly around conserved or repetitive regions.^57^ Combining bait capture enrichment with long-read sequencing technologies (e.g., PacBio, Nanopore), as demonstrated by the target-enriched long-read sequencing (TELSeq)^43^ approach, addresses this limitation. Long-read sequencing data can span entire ARGs and flanking regions, directly revealing colocalization with MGEs without relying on potentially erroneous bioinformatic assemblies.^58^ Initial work with TELSeq used ARG-specific baits and captured flanking MGEs through a “bystander” effect. Further studies explored the utility of different probe sets, comparing ARG-only, MGE-only, and combined ARG-MGE baits.^44^ While performance varied with sample type and enrichment protocol, the combined ARG-MGE probe set was found to be highly effective for comprehensive resistome and mobilome analysis, significantly increasing on-target rates for both ARGs from 0.1% to 2.9% and MGEs from 2.2% to 13.2% in human feces compared to non-enriched sequencing. This ability to directly link ARGs with MGEs provides insights into the transferability of resistance within microbial communities.

Bait capture enrichment is also highly valuable in agricultural and plant sciences, particularly for studying large, complex gene families in organisms with large genomes, like wheat, or for identifying genes in non-model organisms. Resistance gene enrichment sequencing (RenSeq)^59^ was developed to enrich for the large and complex family of NB-LRR disease resistance genes. When applied to the tomato genome, RenSeq successfully increased the number of identified NB-LRRs compared to previous annotations. Likewise, Chen et al.^54^ developed generic-mapping enrichment sequencing (GenSeq) to enrich for ∼2,000 low-copy-number genes anchored to the potato genome. Applying both RenSeq and GenSeq to the wild potato relative *Solanum verrucosum* enabled the identification and mapping of a novel gene conferring broad-spectrum late blight resistance to chromosome 9. This targeted approach mirrors successes seen in plant pathology, where bait enrichment enables the identification and study of pathogens impacting crops, improving detection accuracy and facilitating the analysis of low-copy-number genes.^60^^,^^61^ Bait capture enrichment is also effective for characterizing specific gene families related to crop quality. Jouanin et al.^62^ developed GlutEnSeq to enrich for complex gluten gene families in wheat and related species, achieving ∼10,000-fold enrichment and enabling the study of mutations relevant to celiac disease. The development of such targeted protocols also finds parallels in methods like pathogen enrichment sequencing (PenSeq), which has been used for oomycete pathogens in plant biology.^61^^,^^63^ Bait capture enrichment has been successfully applied to phylogenetics in challenging groups like oomycetes. Nguyen et al.^64^ designed baits for 426 oomycete-specific orthologs and barcoding genes, demonstrating high specificity and successful ortholog recovery even from historical herbarium specimens, paving the way for unlocking genetic information from natural history collections. These examples, listed in Table 3, illustrate the efficacy of bait capture enrichment for targeting genes to facilitate gene discovery, genetic mapping, and phylogenetic studies in agriculturally important organisms and pathogens. Such tools are essential for accelerating breeding programs aimed at goals like resistance gene stacking for durable pathogen resistance or genomic selection for identifying superior animal genetics.^65^

**Table 3:** Overview of selected bait capture enrichment technologies and approaches

Technology	Year	Description
RenSeq	2013	resistance gene enrichment sequencing specifically targeting the NB-LRR gene family in plants for mapping and discovery^59^
MEGaRICH	2017	bait capture enrichment (using MEGARes v1.0 baits) combined with UMIs for improved quantification and detection of rare ARGs and virulence factors in metagenomes^3^
ResCap	2018	platform targeting >78,000 genes associated with resistance (antibiotics, metals, biocides) and plasmid markers^4^
GenSeq	2018	GenSeq enriching for more than 2000 single/low-copy number genes (including COS genes) anchored across the potato genome for mapping studies^54^
CARD-Capture	2019	approach using baits designed from the CARD for resistome analysis in diverse samples (e.g., bacteria, human stool)^41^^,^^56^
GlutEnSeq	2019	enrichment system targeting complex gluten gene families (prolamins) in Triticeae species (e.g., wheat) for variation and mutation studies (∼10,000-fold enrichment)^62^
Oomycete TE	2021	bait set enriching 426 single-copy oomycete-specific orthologs and 3 barcoding genes for phylogenetic analysis, effective on live and herbarium specimens^64^
TELSeq	2022	TELSeq combining bait capture (initially MEGARes v2.0 baits) with long-read sequencing (PacBio) to contextualize ARGs with MGEs^43^
TELSeq II	2024	refinement of TELSeq using ARG-only, MGE-only, or combined ARG-MGE bait sets with long-read sequencing (Nanopore) across different input protocols (XT and XT-HS2)^44^

This table summarizes techniques aimed at enriching specific genes or gene families, such as those conferring antimicrobial resistance (ResCap, MEGaRICH, CARD-Capture, TELSeq), plant disease resistance (RenSeq), general mapping (GenSeq), crop quality (GlutEnSeq), or phylogenetic markers (Oomycete TE). These methods utilize hybridization capture with baits designed from reference databases or genomic resources to increase the detection sensitivity and sequencing efficiency for target genes in complex genomic or metagenomic samples. Integration with long-read sequencing (TELSeq) further enables contextual analysis, such as linking ARGs to MGEs.

## Computational problems in bait capture enrichment

Bait enrichment methods are widely used to isolate and amplify specific genomic regions of interest for further analysis. While highly effective, these techniques introduce several computational challenges. Designing optimal bait sets, managing the computational load required to process large sequencing datasets, and accurately distinguishing between on-target and off-target sequences are all significant hurdles that can impact the precision and efficiency of bait enrichment in genomic research. Addressing these computational issues is essential for advancing the application of enrichment techniques in genomic research.

**Figure 3:**
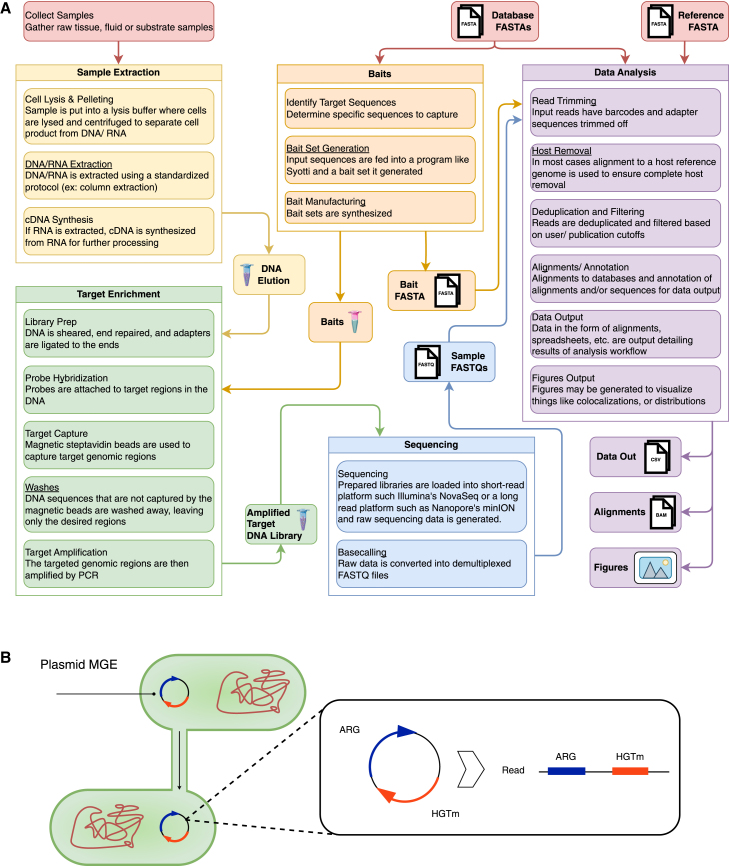
Lab and computational workflow for bait enrichment and ARG-MGE colocalization (A) The diagram outlines each stage of the bait enrichment process, from sample collection through sequencing and data analysis, highlighting the required inputs and outputs at each step. Both experimental procedures and computational steps are encompassed. (B) This figure illustrates horizontal gene transfer of an antibiotic resistance gene (ARG) via a plasmid mobile genetic element (MGE). The plasmid contains both the ARG and horizontal gene transfer machinery (HGTm)—the molecular components required for gene transfer between bacterial cells. ARG-MGE colocalization refers to the physical proximity of resistance genes and mobile genetic elements on the same DNA molecule, which facilitates the spread of antibiotic resistance through bacterial populations.

Figure 3A provides an integrated overview of the laboratory and computational workflows involved in bait enrichment, detailing the key inputs and outputs required at each stage. This work flow illustrates both the experimental and analytical components, underscoring the complexity of the process and the points where computational improvements could enhance overall workflow efficiency.

### Constructing baits

Bait construction is a crucial component of bait capture enrichment, which is done computationally prior to any laboratory work. At the present moment, there are a handful of methods to construct a bait set for a given set of target sequences. These methods include Syotti,^66^ AnthOligo,^67^ CATCH,^67^ MrBait,^68^ BaitsTools,^69^ BaitFisher,^70^ and MetCap.^71^ Table 4 generalizes the steps taken by these algorithms for creating baits.

**Table 4:** Computational workflow for designing bait sequences for bait capture enrichment

Computational step	Description
(1) Segment targets	target regions are divided into smaller overlapping segments that the baits will cover, ensuring complete coverage
(2) Generate candidates	for each segment, candidate bait sequences are generated, typically at length of 60–150 nucleotides
(3) Optimize coverage	the length and overlap of baits is optimized to ensure uniform coverage of the target regions while minimizing redundancy
(4) Filter duplicates	candidate baits are filtered for uniqueness, ensuring they have low homology to off-target genomic regions
(5) GC check	the GC content of the baits is analyzed to ensure it is within an optimal range (usually 40%–60%) to avoid hybridization issues
(6) Generate list	the list of optimized baits that effectively cover the target regions with high specificity and efficiency is compiled

Key steps include segmenting target regions, generating and optimizing candidate baits, filtering for uniqueness and optimal GC content, and compiling a finalized bait list with high specificity and efficiency for the target regions.

MetCap and BaitFisher are two of the first tools designed for the automated and efficient design of capture probes for bait capture enrichment in high-throughput sequencing. MetCap was released in 2015 and is a tool for designing capture probes tailored for metagenomic studies, focusing on targeting conserved and variable genomic regions across multiple species within a microbial community. It works by analyzing input reference sequences or taxa to identify conserved regions that are common across diverse organisms while allowing flexibility for variable regions. MetCap then designs probes that balance coverage and specificity, filtering for optimal hybridization properties like melting temperature and sequence specificity to minimize off-target binding. The resulting probes are optimized for capturing a broad and phylogenetically diverse set of organisms, making MetCap particularly useful for studying complex microbial communities in metagenomic sequencing. BaitFisher starts with a multiple alignment of the target sequences. It clusters the sequences based on Hamming distance and generates a consensus sequence for each cluster, aiming to minimize the maximum Hamming distance within the cluster. The consensus sequence is computed either through an exhaustive brute-force method or a greedy heuristic approximation. BaitFisher then creates a provisional bait set by tiling these consensus sequences with baits. A post-processing step, using a tool called BaitFilter, removes potential issues like baits binding to multiple regions.

In 2018, MrBait and CATCH were released. MrBait works by fragmenting input sequences into overlapping *k*-mers, optimizing probe selection to minimize redundancy while ensuring sufficient coverage, and filtering for thermodynamic properties and specificity to avoid off-target binding. CATCH generates candidate baits by sliding a window over input sequences and uses locality sensitive hashing (LSH) to filter duplicates. Two LSH options are offered: Hamming distance based and minimizer hashing. Candidates are mapped to target sequences using a seed-and-extend approach. A tunable hybridization model determines bait-target matches based on user-defined parameters for mismatch tolerance, exact match requirements, or hybridizing substrings. Redundant baits are filtered by solving a set-cover problem with a greedy heuristic. To avoid overrepresentation of diverse taxa, a weighted optimization adjusts hybridization parameters using a constrained non-linear optimization algorithm, ensuring balanced probe coverage across taxa.

Alanko et al.^66^ were the first to give a computational definition for the problem and demonstrate that the problem is computationally difficult even for really restricted versions of the problem. In light of the hardness results, they develop and implement a heuristic for finding baits, which is referred to as Syotti. Syotti works by first building an FM index^72^ and a generalized suffix array^73^ for input data, bait sequences are initialized to an empty set, and a bit vector is initialized for each sequence, which signifies the covered positions of the bait sequences. Next, a linear scan happens and, when a non-covered position is found, a new bait is added to the set and everything is updated. In summary, multiple bait sets are established, ensuring that all specified regions are covered.^66^

While these methods perform well, there are still areas of future work that can improve bait construction, such as considering the GC content of the baits, designing baits in a manner that avoids contaminants, designing baits to avoid bait-to-bait binding, and designing baits in a manner that avoids off-target binding. While many manufacturers perform quality-control steps to balance the GC content and bait-to-bait binding, many bait-design algorithms do not incorporate these considerations. Next, we discuss these in more detail.

#### Design complexity

Choosing the right genomic regions to target is complex and requires comprehensive knowledge of the genome and the biological questions being addressed. Syotti simplifies this by finding all possible positions for valid baits. Ensuring uniform coverage across all targets is challenging, as some regions may inherently capture more efficiently than others. Syotti designs baits to ensure high coverage.

#### Bait synthesis

Baits need to be highly specific to the target regions without capturing off-target sequences, which requires precise sequence design. Syotti uses a seed-and-extend method that starts with exact matches. The length and nucleotide composition of baits can affect hybridization efficiency and specificity. Longer probes might provide better specificity but may also be more expensive and harder to synthesize. Syotti allows users to choose the length of baits.

#### Hybridization efficiency

Regions with high or low GC content can hybridize inefficiently, leading to uneven coverage. Additionally, secondary structures in both the bait and target DNA can inhibit hybridization.

#### Technical and biological variability

The quality and quantity of input DNA can affect enrichment efficiency. Degraded or insufficient DNA can lead to poor enrichment. Genetic variation, such as single-nucleotide polymorphisms (SNPs) and indels, can affect bait binding and lead to uneven coverage or missed targets. Syotti accommodates up to 30% mismatch to address this issue.

In conclusion, the construction of baits is essential for bait capture enrichment, allowing for the selective capture of specific genomic regions. Various computational tools have been developed to optimize this process, each with distinct approaches to handle challenges like sequence variability, redundancy, and hybridization specificity. While tools like MetCap, Bait Fisher, MrBait, and CATCH offer robust methods for designing baits, further improvements are needed in addressing issues such as GC-content balance, off-target binding, and bait-to-bait interactions, which are critical for ensuring high-quality probe sets and reliable enrichment results. These considerations highlight areas for future work to enhance bait design efficiency and accuracy.

### Deduplicating enriched data

Duplicates refer to sequences that appear multiple times within a dataset but originate from the same source. These can arise in several ways during genomic analysis. One common source is PCR duplicates, which occur during the library preparation step of sequencing. During PCR amplification, the same DNA fragment can be copied multiple times, resulting in identical reads in the sequencing output. These are technical artifacts rather than independent observations.^74^^,^^75^ Another type of duplicate is naturally occurring duplicates, which are sequences genuinely repeated within the genome itself. Certain genes or genomic regions may be present in multiple copies within an organism’s genome, often due to evolutionary processes like gene duplication events.^76^^,^^77^ Sequencing duplicates also occur when the same DNA fragment is sequenced multiple times due to the random nature of sampling during sequencing. This can happen if the sequencing depth is high, leading to multiple reads covering the same genomic location.

To address artificial duplicates, a process called deduplication is used. Deduplication involves identifying and removing duplicate reads from genomic data. This process is essential for improving the quality and accuracy of downstream analyses, such as variant calling, transcript quantification, and structural variant detection. While deduplication might not significantly impact the accuracy of variant detection, it can enhance the annotation of variants and reduce noise in more complex analyses, such as structural variant detection. Hence, computationally handling duplicates effectively is crucial in genomic analysis since it prevents the overestimation of sequence coverage and avoids biases in downstream analyses. Bait enrichment adds complexity to the deduplication process since it is designed to increase the coverage of specific genomic regions, which can lead to a high number of redundant reads. This makes it challenging to identify and remove duplicates accurately without losing true signal. Enrichment methods can also introduce biases, such as uneven coverage across target regions, complicating the distinction between true duplicates and genuine reads representing different molecules but covering the same region. Additionally, multiple rounds of PCR amplification during enrichment can create duplicates that are technical artifacts rather than biological duplicates, further complicating the deduplication process. While amplification bias is common to all PCR-based workflows, this source of bias may be exacerbated in data generated from bait enrichment due to the additional post-enrichment amplification cycles combined with the high proportion of library originating from targeted sequences. It is also important to consider that, for bait enrichment applications intended for short-read sequencing, library preparation protocols are designed to yield a narrow distribution of post-enrichment DNA fragment lengths optimized for the sequencing platform. The use of short reads in bait enrichment can lead to multiple short fragments aligning to the same region, making it difficult to distinguish between technical duplicates and legitimate overlapping reads. Finally, the reduced complexity of bait capture enrichment increases the likelihood of sequencing the same molecule multiple times, complicating deduplication.

To address these challenges, advanced deduplication algorithms and additional data, such as unique molecular identifiers (UMIs), are often employed. UMIs help in accurately identifying and removing duplicates by tagging each original DNA molecule with a unique sequence before amplification. The challenges posed by bait capture enrichment underscore the need for more accurate and efficient methods of deduplication to ensure high-quality genomic data analysis. However, even when UMIs are used, they are not always able to be used for deduplication due to sequencing errors. For example, in TELSeq,^43^ UMIs were siloed in the sequencing data and, thus, the PacBio sequence reads were deduplicated by clustering by length, then performing pairwise alignment, and taking a consensus. There are opportunities for further development in this area.

To summarize, managing duplicates in genomic data is essential for ensuring accurate and reliable downstream analyses, particularly in contexts like variant calling and structural variant detection. While deduplication processes and technologies such as UMIs have improved the identification and removal of duplicates, challenges remain, especially in complex scenarios involving bait enrichment and short-read sequencing. Addressing these challenges requires ongoing advancements in deduplication algorithms and methods that account for both technical artifacts and biological variability.

### Deciphering colocalizations

Colocalization in genomics refers to the physical proximity of different genetic elements within the genome, such as genes and regulatory elements like enhancers and promoters. Identifying these colocalizations is critical for understanding functional interactions within the genome. For instance, if a gene and its regulatory enhancer are colocalized in the three-dimensional space of the nucleus, it suggests that the enhancer may directly influence the gene’s expression. Detecting such 3D chromatin interactions typically requires specialized techniques like Hi-C,^78^ ChIA-PET,^79^ or Capture-C,^80^ which can capture the spatial organization of the genome. In TELSeq,^43^ colocalizations are identified by locating ARGs that are adjacent to an MGE on the same read. Colocalizations involving the same gene pairs are considered distinct if they occur at different distance thresholds, such as 100 or 350 bp between the genes. This method of identifying colocalizations highlights how small variations in read alignment can reveal significant biological relationships between genes that are close together on the genome, potentially indicating functional gene clusters or horizontal gene transfer events (Figure 3B).

Using bait capture enrichment with short-read sequencing can pose challenges in identifying colocalizations. Short-read sequencing typically generates reads that are only 150–250 bp long, making it unlikely for a single read to capture entire genes—often over 200 bp long—along with additional colocalized genes or elements. Genome assembly may be effective in producing contigs—contiguous DNA sequences assembled from overlapping reads—that flank colocalizations but there is risk of creating false colocalizations due to the inherent challenge in assembling short reads unambiguously.^81^ A more effective approach involves analyzing colocalizations on assembled scaffolds or through long-read sequencing, where extended reads provide a more comprehensive view of gene proximity and interactions.

What are referred to as “safe and complete” genome assemblers offer a promising solution to the assembly challenges that can compromise colocalization studies.^82^ A safe assembler is defined as an algorithm distinguished by the rigorous guarantee of only outputting sequences that are definitely part of the original genome while not missing any regions that could be confidently assembled.^83^ Unlike standard assemblers that rely on heuristics and optimization, potentially introducing misassemblies or missing true sequences, safe and complete assemblers prioritize accuracy over contiguity.^84^ This conservative approach is particularly valuable for colocalization analysis, as it reduces the risk of false colocalizations that arise from assembly artifacts while ensuring that genuine gene proximities are not overlooked due to assembly gaps.^85^ By providing a more reliable genomic foundation, these assemblers enable researchers to distinguish between authentic biological colocalizations and those artificially created by assembly errors, ultimately supporting more accurate and meaningful colocalization findings in complex genomic regions. However, this conservative approach comes with trade-offs, as safe and complete assemblers typically produce more fragmented assemblies with shorter contigs to avoid potentially incorrect connections and may miss complex or repetitive genomic regions where colocalizations might occur, potentially limiting the scope of discovery.

Another limitation of bait capture enrichment is the loss of broader genomic context. By focusing on isolated regions, bait capture may overlook the relative spatial positions and interactions that are essential for identifying colocalizations.^86^ For instance, colocalization often depends on how elements are organized in the three-dimensional architecture of the genome. Isolating these regions disrupts the natural interactions and makes it harder to detect functional relationships between colocalized elements.^87^

### Detecting variants from enriched data

Variant construction, the process of identifying and characterizing genetic variations within a genome, faces unique challenges when dealing with bait-enriched sequencing data. While bait enrichment strategies allow researchers to focus on specific genomic regions, they introduce several technical hurdles that can impact the accuracy and completeness of variant detection. Bait enrichment often results in uneven coverage across the targeted regions.^7^ The degree of this coverage uniformity can vary substantially depending on the application and sample complexity. While manufacturers may provide expected uniformity metrics for well-defined targets like human exomes, uniformity is often less predictable and more challenging to achieve in highly complex inputs such as metagenomic samples.^88^ Some areas may be overrepresented due to preferential capture, while others might be underrepresented or missed entirely. This uneven coverage can significantly complicate variant detection, especially in low-coverage regions where confidence in variant calls is reduced.^89^ Additionally, incomplete capture due to inefficient probe design or suboptimal conditions can leave gaps in the sequencing data, making comprehensive variant identification difficult.

Off-target sequences are frequently captured during the enrichment process, introducing noise and complicating analysis.^90^^,^^91^ Highly repetitive regions, structural variants, and areas with complex architectures (such as those with high GC content) remain particularly challenging to sequence and analyze accurately.^92^ Detecting structural repeats and copy number variants (CNVs) is especially difficult because the enrichment process may either fail to capture these regions adequately or introduce artifacts that obscure genuine variant signals.^93^

The enrichment process often involves PCR amplification, which can introduce biases and artifacts. Issues like mismatches in primer design, suboptimal PCR conditions, and other technical factors can lead to erroneous amplification, either masking true variants or producing false positives. These biases can distort the data by skewing copy number estimates or causing misalignment, particularly when primers are designed using an incomplete or mismatched reference genome.

Computational analysis of enriched data is inherently limited by the scope and quality of the input sequences. When working with bait-enriched sequencing, the analysis is restricted to the regions captured, leaving any variants outside these regions undetectable. Even within the targeted regions, complex bioinformatic pipelines are needed to correct for biases and artifacts, requiring substantial computational resources and sophisticated algorithms.

Addressing these challenges may require a combination of laboratory improvements and computational innovations. One approach is to use a reference genome to create a pseudo-“mask” representing the uncaptured portions of the genome. However, this method introduces bias since these masked regions lack real sequencing depth and variability, which could lead to inaccurate conclusions. Incorporating pangenomic references that aggregate multiple genomes from diverse populations might reduce ethnic biases and improve variant detection across different genomic backgrounds. While promising, this approach still struggles to fully capture the complexity of structural variants and repeat regions.

Ultimately, many of these challenges may be addressed more effectively in the laboratory than through computational solutions alone. Refining probe design, optimizing PCR conditions, and improving capture protocols could mitigate some of the technical limitations currently hindering variant detection in bait-enriched sequencing data.

### Modeling off-target binding

While bait capture enrichment generally improves the recovery of difficult-to-sequence regions, allowing for efficient and accurate analysis,^94^ bait enrichment can still lead to substantial amounts of off-target sequences making it into the data, particularly when homologous sequences are prevalent in the sample used for probe design.^95^ Off-target sequences, in the context of bait enrichment, refer to valid but undesired data captured during the enrichment process that do not correspond to the intended target regions of a reference genome or database. These off-target sequences can be classified into a few categories. First, true negatives (non-specific binding): off-target sequences that originate from the sample itself but bind to non-target regions due to sequence similarity. This often occurs when there are homologous regions within the genome or across closely related species. The presence of these sequences can lead to the incorrect characterization of genomic regions and introduce noise in variant detection. Second, false positives (contaminants binding to target regions): sequence contamination can occur when unrelated sequences from different organisms or experimental sources bind to the intended target regions. These sequences mimic genuine target signals, leading to incorrect conclusions about the presence or abundance of particular genomic elements. Third, false negatives (homologous sequences misaligned): sequences that are correctly captured but homologous to off-target regions can result in incorrect alignments during analysis. In cases where homologous regions share significant sequence identity, it can be challenging to distinguish between true target signals and artifacts, leading to potential misinterpretation of results.

Strict bioinformatic filtering can mitigate some of these issues by establishing stringent criteria for sequence alignment and identification. For example, Bossert et al.^96^ suggest using thresholds such as a minimum sequence identity of 82%–85% across an overlap region of 80% to score a positive hit. These parameters help to differentiate between true and false signals in complex datasets, especially when dealing with bait enrichment designed for specific organisms that may inadvertently capture off-target sequences from related taxa. Further computational models can refine the trade-off between on-target and off-target hybridization. For instance, Matveeva et al.^97^ demonstrated that sequence characteristics, such as GC content and secondary structure, can be used to model and predict the hybridization efficiency of oligoprobes. This modeling approach helps to optimize probe design by accounting for the inherent trade-offs between maximizing target capture while minimizing off-target effects. Additionally, tools like Hotz et al.^98^ highlight the limitations of single-alignment strategies when dealing with highly homologous regions. In these cases, algorithms that rely solely on a single best alignment can miss important nuances in the data, such as low-complexity regions or subtle sequence variations that might be critical for accurate characterization.

Finally, off-target effects are not only a computational challenge but also a biological one. Even when stringent computational filters are applied, certain regions of the genome, like repetitive elements or regions with high sequence similarity, remain prone to capturing off-target sequences. Improving bait design to avoid these regions, while still providing comprehensive coverage of the targets, is an ongoing area of research. Incorporating machine-learning models that predict off-target risks based on sequence features and integrating these predictions into bait design algorithms could offer a more proactive approach to minimizing off-target capture.

In summary, modeling and mitigating off-target binding requires a multi-faceted approach that combines improved probe design, stringent bioinformatics filtering, and sophisticated computational models. Advances in this area are crucial for enhancing the accuracy and reliability of bait enrichment experiments, particularly as the complexity and scale of genomic studies continue to grow.

## Conclusions and future perspectives

In this review, we explored the use, applications, and limitations of bait capture enrichment methods for genomic sequencing, focusing on applications for enriching distinct biological targets such as bacteria, viruses, and genes. While bait capture enrichment offers significant advantages, particularly in terms of cost-efficiency and increased sequencing depth for regions of interest, it also presents notable challenges that impact the accuracy and scope of the resulting data. These challenges lead to interesting avenues for future work. Here, we discuss some of these in more detail.

### Addressing input limitations and technical challenges

One challenge of bait capture enrichment is the typical requirement for substantial starting DNA mass. Most standard protocols necessitate relatively high DNA inputs of at least 100–500 ng, which can be prohibitive when working with low-biomass samples.^99^ To address this limitation, low-input capture kits^100^ have been developed that reduce requirements to as little as 10 ng of input DNA through optimized adapter concentrations, enhanced ligation conditions, and longer bait sequences for improved binding specificity.^15^

However, low-input approaches introduce additional technical considerations. The reduced starting material necessitates additional PCR amplification cycles to generate sufficient library yield, which elevates duplication rates and increases the risk of polymerase-induced errors.^101^ Each PCR cycle introduces an estimated 0.1%–1% error rate that compounds with increasing cycle number.^102^ Low-input hybridization protocols also exhibit higher fold-80 base penalty scores and amplified GC bias effects, particularly in genomic regions with <30% or >70% GC content.^103^

### Computational advances in probe design

Beyond addressing input limitations, the future of bait capture enrichment also depends on computational advances in probe design and integration with emerging sequencing technologies. Current methods often rely on overly simplistic approaches that consider only sequence similarity and total sequence coverage, creating opportunities for improvement. Next-generation bait-design algorithms must incorporate GC-content balancing and comprehensive off-target prediction, as pseudogenes and regions of high homology can create opportunities for off-target binding that reduce on-target data yield.^104^

Future algorithms should integrate thermodynamic modeling, secondary structure predictions, and machine-learning approaches trained on empirical hybridization data to improve design accuracy. Many existing tiling methods prioritize exhaustive coverage but lack rigorous post-processing steps to filter baits with high similarity to non-target regions, increasing cross-hybridization risk. While tools like BaitFilter^70^ attempt to remove problematic baits *post hoc*, not all workflows incorporate this critical step. More sophisticated approaches for targeting highly diverse sequences, such as k-mer clustering methods used by ProbeTools,^105^ represent promising alternatives to simple tiling strategies. Additionally, longer baits can capture larger DNA fragments, enabling the creation of “sparse” bait sets with wider spacing that still achieve complete sequence capture.^106^

### Integrated optimization frameworks

Improved bait-selection metrics are equally important for advancing the field. While many modern bait-design methods already incorporate capture uniformity optimization and specificity considerations during the design phase, there remains a need for more comprehensive, integrated frameworks that simultaneously optimize uniformity, specificity, and efficiency through unified quantitative metrics rather than addressing these objectives separately or through *post hoc* empirical optimization.^107^ Advanced algorithms must balance competing objectives, including maximizing coverage while minimizing off-targets, optimizing uniform coverage while maintaining cost-effectiveness, and designing for broad taxonomic coverage while ensuring adequate specificity. Furthermore, refining bioinformatic workflows to handle enriched data nuances is essential through improved deduplication methods, enhanced variant calling that accounts for coverage biases, and better detection of genomic colocalizations.^108^^,^^109^

### Integration with long-read sequencing technologies

Building on these computational advances, long-read sequencing with bait capture enrichment represents a particularly promising direction that addresses many current limitations. This integration enables detection of complex structural variants down to 50-bp resolution that escape short-read detection,^110^ megabase-scale haplotype phasing that resolves compound heterozygosity,^111^ and direct access to previously intractable genomic regions including centromeres, segmental duplications, and highly repetitive sequences.^112^ Moreover, this approach could provide native detection of DNA methylation patterns including 5mC and 6mA modifications without bisulfite conversion,^113^ eliminate PCR amplification biases and GC-content artifacts through single-molecule sequencing,^114^ and enable direct allele-specific analysis of captured loci including compound heterozygous variants without requiring family-based phasing approaches.^115^^,^^116^

### Complementary and alternative enrichment strategies

In parallel with these technological advances, alternative enrichment strategies are emerging that complement traditional bait capture approaches. Concurrently, optimizing laboratory protocols for challenging samples, including low-input, degraded, or environmental specimens,^117^ and developing reverse enrichment strategies through depletion methods will expand the applicability of bait capture. These methods, conceptually akin to a “reverse bait capture,” use probes or other biochemical methods to remove highly abundant, unwanted sequences, allowing the target sequences in the supernatant to be sequenced, or enable real-time computational rejection of unwanted reads during sequencing.

Adaptive sequencing represents another emerging complementary enrichment strategy that leverages real-time computational rejection of off-target sequences, achieving 8- to 12-fold improvements in target coverage compared to traditional post-sequencing filtering.^118^ This approach can leverage compressed pangenomic indexes using RLZ, FM-index, and MEM-based methods to improve real-time decision efficiency, although the real-time basecalling required for adaptive sequencing demands substantial computational resources that may limit broader applicability.

While adaptive sequencing provides valuable target enrichment with flexibility and rapid implementation, it generally achieves lower absolute coverage and uniformity compared to hybridization capture methods.^106^^,^^119^ Critical optimization considerations include fragment length relative to target regions—particularly relevant for metagenomic applications where ARGs may span only 2–5 kb—as excessive fragment lengths lead to inefficient pore utilization and increased pore blocking.^120^ For ARG detection specifically, adaptive sequencing demonstrates strength in identifying plasmid-encoded genes with high reference similarity,^121^ while hybridization enrichment remains superior for low-abundance or divergent variants.^122^

### Future integration and optimization

The integration of adaptive sequencing with existing bait capture workflows presents both opportunities and technical challenges, requiring careful optimization to avoid diminishing returns and technical incompatibilities, particularly regarding library-preparation modifications and fragment-size balance. Future developments in pangenomic graph representations and distributed indexing approaches will be essential for adapting compressed indexes from eukaryotic contexts to the complex landscape of dispersed bacterial ARGs across diverse genomes.^123^^,^^124^ As computational capabilities advance and real-time sequencing technologies mature, adaptive sequencing will likely serve as a valuable complement to traditional enrichment methods rather than a replacement, with optimal applications determined by specific project requirements, sample characteristics, and the balance between rapid flexible enrichment versus comprehensive uniform coverage. These advances will collectively drive the development of standardized enrichment panels for clinical diagnostics and infectious disease surveillance,^125^ expanding the practical applications of these methods across diverse research and clinical contexts.

### Broader applications

Despite these challenges, bait capture enrichment remains a powerful tool in specific contexts where high-depth sequencing of targeted regions is critical. For example, in evolutionary biology and agricultural genomics, bait capture allows for the efficient analysis of conserved or variable genomic regions across multiple species by increasing the representation of target sequences and improving sequencing efficiency, enabling deeper insights into trait development and biodiversity.

Given its ability to selectively enrich target sequences prior to sequencing, it is also likely to be well suited for other environmental applications, including the analysis of environmental DNA (eDNA) and wastewater samples.^126^ These sample types are typically characterized by high levels of background DNA originating from diverse microbial, plant, animal, and human sources, as well as degraded or fragmented nucleic acids resulting from environmental exposure. As a result, the relative abundance of target sequences, such as pathogen genomes or biomarkers, is often exceedingly low.^127^ Bait capture has the potential to substantially increase the proportion of on-target reads, thereby improving sensitivity and enhancing the resolution of downstream analyses. This enables the reliable detection of low-frequency targets that would otherwise remain undetected. In eDNA studies, this facilitates comprehensive biodiversity assessments, monitoring of invasive or endangered species, and longitudinal tracking of ecological changes.^128^ In wastewater-based epidemiology, bait capture enrichment enables the detection of circulating pathogens and identification of emerging variants of concern.^129^

The continued development of computational tools, integration with long-read technologies, and optimization of protocols for challenging samples will further enhance the utility and accessibility of these methods across diverse research applications, positioning it as an increasingly valuable tool for high-resolution environmental surveillance and public health monitoring in complex and low-quality samples.

## Acknowledgments

This work was supported by a grant from the 10.13039/100000060National Institute of Allergy and Infectious Diseases (NIH NIAID) (1R01AI173928-01A1). Icons used in Figure 1 were obtained from BioIcons (https://bioicons.com) under Creative Commons licenses; specifically, the nanopore_sequencing and genomesequencer-2 icons by DBCLS (https://togotv.dbcls.jp/en/pics.html, CC BY 4.0), the Illumina_sequencing_colorcalling icon by Bjoern Usadel (usadellab.org, CC BY 4.0), the Beaker_water icon by nUll (CC0), and the blood_sample icon by Marcel Tisch (https://twitter.com/MarcelTisch, CC0).

## Author contributions

J.E.B., investigation, visualization, writing – original draft, and writing – review & editing; K.J.N., writing – review & editing; N.N., funding acquisition and writing – review & editing; C.B., funding acquisition and writing – review & editing.

## Declaration of interests

The authors declare no competing interests.
